# Outcomes and survival following thoracic endovascular repair in patients with aortic aneurysms limited to the descending thoracic aorta

**DOI:** 10.1186/s13019-023-02285-3

**Published:** 2023-06-20

**Authors:** Katharina Fankhauser, Isaac Wamala, Adam Penkalla, Roland Heck, Robert Hammerschmidt, Volkmar Falk, Semih Buz

**Affiliations:** 1 Deutsches Herzzentrum der Charité (DHZC), Department of Cardiothoracic and Vascular Surgery, Augustenburger Platz 1, 13353 Berlin, Germany; 2 Charité – Universitätsmedizin Berlin, Freie Universität Berlin, Humboldt-Universität zu Berlin, and Berlin Institute of Health, Augustenburger Platz 1, 13353 Berlin, Germany; 3 German Center for Cardiovascular Research (DHZK), Partner Site Berlin, Berlin, Germany; 4 Translational Cardiovascular Technologies, Institute of Translational Medicine, Department of Health Sciences and Technology, Swiss Federal Institute of Technology (ETH), Zurich, Switzerland

**Keywords:** TEVAR, Thoracic aneurysms, Risk factors

## Abstract

**Background:**

Thoracic endovascular aortic repair (TEVAR) is a well-established therapy for descending aortic aneurysms (DTA). There is a paucity of large series reporting the mid- and long-term outcomes from this era. The main aim of this study was to evaluate the outcomes of TEVAR with regards to the effect of aortic morphology and procedure-related variables on survival, reintervention and freedom from endoleaks.

**Methods:**

In this retrospective single center study, we evaluated the clinical outcomes among 158 consecutive patients with DTA than underwent TEVAR between 2006 and 2019 at our center. The cohort included 51% patients with device landing zones proximal to the subclavian artery and 25.9% patients undergoing an emergent or urgent TEVAR. The primary outcome was survival, and secondary outcomes were reintervention and occurrence of endoleaks.

**Results:**

Median follow-up was 33 months [IQR 12 to 70] while 50 patients (30.6%) had longer than 5-year follow-up. With a median patient age of 74 years, post-operative Kaplan Meyer survival estimates were 94.3% (95%CI 90.8–98.0, SE 0.018%) at 30 days, 76.4% (95%CI 70.0–83.3, SE 0.034%) at one year and, 52.9% (95%CI 45.0–62.2, SE 0.043%) at five years. Freedom from reintervention at 30 days, one year, and five years was 92.9% (95%CI 89.0–97.1, SE 0.021%), 80.0% (95%CI 72.6–88.1, SE 0.039%), and 52.8% (95%CI 41.4–67.4, SE 0.065%), respectively. On cox regression analysis greater aneurysm diameter, and the use of device landing zones in aortic regions 0–1 were associated with an increased probability of all-cause mortality, and with reintervention during follow-up. Independent of aneurysm size undergoing urgent or emergent TEVAR was associated with higher mortality risk for the first three years post-operative but not on long-term follow-up.

**Conclusions:**

Larger aneurysms and those requiring stent-graft landing in aortic zones 0 or 1, are associated with higher risk for mortality and reintervention. There remains a need to optimize clinical management and device design for larger proximal aneurysms.

**Supplementary Information:**

The online version contains supplementary material available at 10.1186/s13019-023-02285-3.

## Introduction

Thoracic endovascular repair (TEVAR), when anatomically feasible, is currently the preferred management modality for descending thoracic aortic aneurysms (DTA), except for patients with connective tissue disease [1, 2]. In comparison to patients who receive open repairs, patients undergoing TEVAR have improved early survival and lower rates of paraplegia and perioperative morbidity [3–6]. Mid-term survival after endovascular and open DTA repair are similar, but TEVAR tends to show higher reintervention rates [5, 7].

The goal of TEVAR is to achieve a good primary seal that completely excludes the aneurysm during the first intervention, with minimal procedure related complications, and that promotes durable aortic remodeling. Anatomic characteristics reported to impact the outcome after TEVAR include the device landing zones (LZ), the length and diameter of the aneurysm, the thrombus volume and the tortuosity of the aorta [8]. The past decade has seen increamental refinements in stent-graft design, delivery systems as well as new improved image-based planning tools for TEVAR. At the same time, indications have shifted to include also patients with more challenging landing zones, including larger and more proximally localized aneurysms.

These rapid developments demand a constant evaluation to identify areas that remain challenging and inform further innovation. The aim of this study was to identify patient and procedure related factors that affect mid-term outcomes following TEVAR for isolated descending thoracic aneurysms in the current practice.

## Patients and methods

### Ethic statement and study design

This single center retrospective study was reviewed and approved by the local ethics committee (Ethics approval number EA2/103/20).

### Patients

**Fig. 1:**
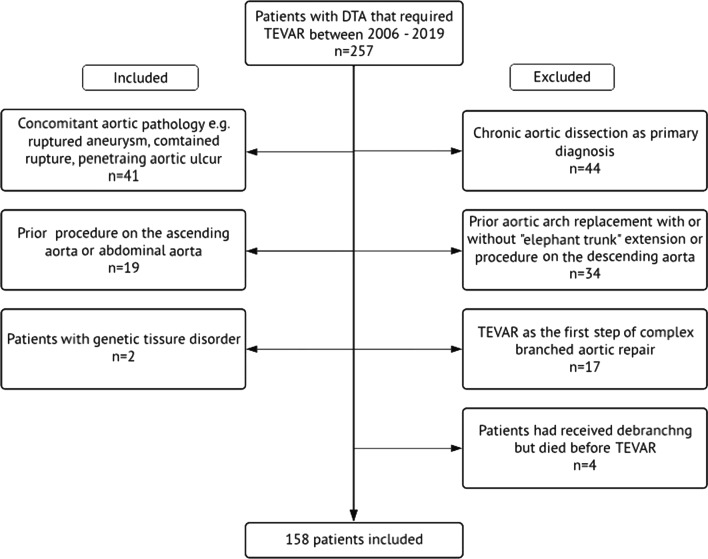
Patient selection

275 patients that underwent TEVAR as the initial intervention for DTA at our institution between January 2006 and November 2019 were screened. As shown in the flow chart in Fig. 1, patients with whose aneurysms extended to the abdominal aorta (thoracoabdominal aneurysms) were excluded. Patients with primarily chronic aortic dissections were also excluded. In addition, patients with prior interventions at either the proximal or distal landing zones were excluded. This was for example patients with prior interventions on the aortic arch, or proximal descending aorta (including TEVAR and open repair with or without 'elephant trunk’). However, prior aortic interventions remote from the aneurysm and remote from the device landing zone (ascending aorta or distal infrarenal aorta) were not exclusion criteria and these were included. A total of 158 patients met the inclusion criteria.

### Follow-up

Survival status was determined by cross-referencing with the civil registry. Clinical follow-up was conducted via review of the clinical notes made during recent post-operative surveillance visits in our outpatient clinic, or directly by patient phone calls for those without a recent outpatient visit.

### Clinical approach

Patients presenting with isolated thoracic aortic aneurysms are considered for TEVAR if there is an adequate landing zone of at least 25 mm both proximally and distally, or if a landing zone can be created by adjunctive debranching. We aim for 15–20 percent diameter-oversizing as measured at the device landing zones. If carotid-subclavian bypass is required for elective TEVAR it is performed in the same sitting, however in cases of urgent TEVAR carotid-subclavian bypass can be performed a few weeks after TEVAR or if the patient is asymptomatic revascularization can be differed.

Postoperative blood pressure management is a key adjuvant therapy. We aim to keep the blood pressure normotensive. All patients received a postoperative CTA prior to discharge and then regular surveillance and follow-up CT scans in our out-patient clinic at one month, six months, one year and then yearly. Our follow up is guided by the ESC guidelines for surveillance after TEVAR [1].

### Morphological parameters

The baseline morphological characteristics of the DTA were assessed from the pre-operative computed tomography angiography (CTA) images, using Aquarius iNtuition (Terarecon—Ver.4.4.13.P5) software. Points 1 to 5b along the centerline of the aorta were assigned to mark the proximal and distal LZ, as well as the aneurysm itself. This way each measurement could be done in a standardized manner, allowing comparison between patients. The points were:

- P1 was the proximal limit of the proximal LZ as marked by the takeoff of the subclavian artery, common carotid artery or brachiocephalic trunk, depending on the surgical strategy and landing zone.
- P2 was the proximal end of the aneurysm.
- P3 was the distal end of aneurysm.
- P4 was distal limit of the distal LZ as marked by the celiac artery takeoff or otherwise by an additional distal aneurysm.
- P5a was a point 30 mm proximal to P2 as proximal limit of the proximal LZ in cases where the distance between P1 and P2 exceeded 30 mm.
- P5b is a point 30 mm distal to P3 as distal limit of the distal LZ in cases where the distance between P3 and P4 exceeded 30 mm.

The morphological characteristics of the aneurysm measured were length of the aneurysm, maximal diameter, total volume and proportion of thrombus. LZ characteristics measured were diameter, area, curvature, and differential length along the centerline, inner or outer curvature of the LZ. Ellipticity was estimated by the ratio of maximal and minimal diameter. Diameter, area and ellipticity at the proximal and distal LZ were measured at the proximal and distal end of the LZ and averaged. Proximal landing zones were classified as described by Ishimaru et al. [9]. Aortic coverage was measured as the ratio of stent length and aortic length from the aortic annulus to the bifurcation in the postoperative CTAs.

### Endoleaks

An endoleak was any radiological evidence of blood outside the stent-graft and or perfusion of the aneurysm sac. The presence or absence of early post-operative endoleaks was determined from the first postoperative CTAs taken during the first three months after TEVAR, as classified and reported by a radiologist experienced in post-interventional aortic imaging. Follow-up CTAs were reviewed to determine the progression of the endoleaks or the late development of new endoleaks.

### Statistical Analysis

**Fig. 2:**
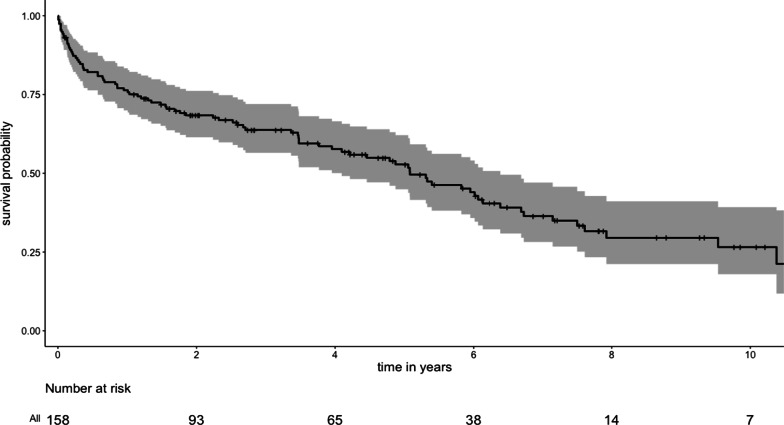
Kaplan–Meier estimate of overall survival

Primary outcome was survival after TEVAR and secondary outcomes were the necessity for aortic reintervention and the occurrence of endovascular leaks. Categorical data was presented as numbers and percentages. Continuous parameters were described as mean ± standard deviation (SD) or medians and interquartile range [IQR] depending on the data distribution type. Kaplan–Meier estimates were used for survival and reintervention analysis and reported with 95% confidence interval (95% CI) (Fig. 2). Univariable cox regression was conducted to evaluate factors related to survival. Variables significant in univariable analysis were used for multivariable cox regression. We limited the number of variables in the multivariate analysis to the number of events according to one-in-ten rule. A competing risks time-to-event analysis was conducted to determine the risk for reintervention, with death as a competing risk, using the mstate package version 0.3.1 in R, as described by de Wreede et al*.* [10] and the results from the transition-specific cox regression analysis were then reported with the corresponding sub-distribution hazard ratio (SHR). Wilcoxon signed-rank tests were used for group comparisons between patients with and without postoperative endoleaks as regards their proximal and distal LZ morphologic parameters, respectively. For morphological parameters found to be significantly different across groups, a calculation using Youden’s index for the “cut-off” value separating the groups was made. This analysis included all endoleaks that were detected up to six months after TEVAR. We determined Kendall rank correlation coefficient to evaluate between time and patients’ and procedural variables. All statistical analysis was conducted in R software.

## Results

### Patients

Patient characteristics are summarized in Table 1. The median patient age was 74 years [IQR 69 to 78]. Forty-three (27.2%) patients presented with acute aortic complications and needed urgent or emergent TEVAR. Median Euroscore II was 3.1 [IQR 1.8 to 4.8] and median aneurysm diameter was 63.7 mm [IQR 58.7 to 74.1]. All aneurysms were of degenerative etiology except in two patients (1.3%), found to have connective tissue disease. In one of these patients Marfan syndrome was confirmed only after TEVAR, and one patient with Loeys-Dietz syndrome, was considered for TEVAR because of a prohibitively high risk for open surgery. Both patients were alive and had not required reintervention at six and five years of follow-up, respectively.

**Table 1:** Patient characteristics and operative details

Patients characteristics (n = 158)	n(%)I mean ± SD or median [IQR]
Age (years)	74 [69,78]
Male (%)	105 (66.5%)
BMI (kg/m^2^)	25.8 ± 4.7
Acute aortic syndrome	43 (27.2%)
Ruptured aneurysm	35 (22.2%)
Contained rupture of aneurysm	3 (1.9%)
New onset penetrating ulcer	3 (1.9%)
With increasing back pain	2 (1.2%)
LVEF (%)	55 [50; 60]
Coronary artery disease	72 (45.6%)
NYHA IV	8 (5.5%)
Atrial fibrillation or flutter	23 (14.6%)
Preoperative creatinine level (mg/dL)	1.00 [0.79, 1.40]
Preoperative EGFR (mL/in/1.73m^2^)	72.0 ± 29.7
Prior stroke	6 (3.8%)
Prior procedure on aorta	19 (12.0%)
Logistic Euroscore	14.7 [9.1, 25.0]
Euroscore 2	3.1 [1.8, 4.8]
Maximum aneurysm diameter (mm)	63.9 [58.7, 74.1]
Aneurysm thrombosed part (%)	48.7 [33.3, 60.5]
*Operative details*	
Duration of TEVAR (min)	113 [68, 160]
Percutaneous vascular access	150 (94.9%)
Proximal landing zones	
0	3 (1.9%)
1	6 (3.8%)
2	72 (45.6%)
3	51 (32.3%)
4	26 (16.5%)
LCCA-LSA or debranching*	70 (44.3%)
Stents combined length (mm)	250 [164, 367]
Aortic coverage (%)	36.6 [28.0, 44.8]
Distance from distal stent to coeliac trunk (mm)	62 [30, 111]
Contrast used (ml)	100 [75, 149]
Dose area product (µg/m^2^)	18,512 [9716, 33717]

*Prior or simultaneously to TEVAR; BMI = body mass index, COPD = chronic obstructive pulmonary disease, LZ = device landing zone, EGFR = estimated glomerular filtration rate, LVEF = left ventricular ejection fraction, NYHA = New York Heart Association

### Procedural details

Median duration of the procedure was 113 min [IQR 68 to 160]. Proximal landing of the thoracic endograft was proximal to the left subclavian artery (aortic zones 0–2) in 81 (51.2%) patients. Among these, strategies for preparing an optimal landing zone in the aortic arch included carotid-subclavian bypass (LSAA-LCA) (n = 61), total debranching (n = 6), partial debranching (n = 3), the use of a stent graft with scallop or chimney stent for the LSA (n = 4) or covering the LSA without revascularization (n = 7). Seventy patients (44.3%) received multiple stents. Evita stent-graft (JOTEC GmbH, Germany) were implanted in 94 patients (59.5%) and Relay stent-graft (Bolton Medical, Inc.) in 59 patients (37.3%). 5 patients (3.2%) received Zenith stent-graft (Cook Medical, Inc.). We did not use cerebrospinal fluid drainage catheters in any patient. Median proximal and distal oversizing was 20.6% [14.1; 26.8] and 20.0% [11.9, 28.1], respectively (Table 2). Our approach for oversizing has remained unchanged over the years and is consistent with society guidelines [1]. We have tended to treat more patients with more proximal landing zones over time. 2006 to 2010 41% of the patients had a landing zone proximal to the LSA, 2011–2014 46% of the patients and after 2015 58% had landing zones proximal to the LSA.

**Table 2:** Morphological characteristics on preoperative CTA

Aneurysm	All patients (n = 158)	Patients with primary endoleak (n = 46)	Patients without endoleak (n = 99)	*p*-value^1^
Length (mm)	122 [52, 191]	166.0 [85.2, 198.5]	103.0 [42.5, 185.0]	0.045
Maximal diameter (mm)	63.9 [58.7, 74.1]	65.7 [58.9, 78.8]	63.4 [58.5, 72.6]	0.14
Total volume (cm^3^)	283 [110, 485]	375.0 [233.0, 551.0]	259.0 [74.7, 430.5]	0.025
Thrombosed part of aneurysm (%)	48.7 [33.3, 60.5]	46.0 [38.4, 59.8]	48.0 [28.6, 60.5]	0.62
Thoracic aorta tortuosity	3.5 [3.0, 4.0]	3.55 [3.17, 4.09]	3.48 [2.91, 3.91]	0.22

Proximal landing zone	All patients	Patients with type Ia endoleak (n = 8)	Patients without typ Ia endoleak (n = 138)	*p*-value^1^
Length on centerline (mm)	30.0 [20.5, 62.1]	26.5 [19.8, 50.3]	30.1 [20.0, 59.9]	0.59
Length on inner curvature (mm)	22.5 [13.9, 42.4]	19.6 [11.3, 32.2]	22.6 [13.9, 41.5]	0.47
Length on outer curvature (mm)	44.8 [29.6, 76.4]	32.2 [24.9, 62.9]	44.7 [28.6, 76.0]	0.37
Curvature	0.26 [0.18, 0.39]	0.34 [0.21, 0.49]	0.25 [0.17, 0.38]	0.31
Cllipticity index of aorta	1.19 [1.12, 1.30]	1.16 [1.11, 1.19]	1.20 [1.12, 1.30]	0.24
Oversizing (diameter) (%)	20.6 [14.1; 26.8]	13.5 [11.6, 18.6]	20.8 [14.6, 26.9]	0.11
Oversizing (area) (%)	54.5 [41.6, 70.1]	42.9 [37.0, 47.6]	54.8 [41.7, 72.3]	0.066

Distal landing zone	All patients	Patients with typ Ib endoleak (n = 4)	Patients without typ Ib endoleak (n = 140)	*p*-value^1^
Length on centerline (mm)	94.3 [57.2, 170.3]	101.6 [76.8, 131.5]	91.9 [54.9, 166.8]	0.94
Curvature	0.19 [0.10, 0.32]	0.30 [0.20, 0.39]	0.19 [0.11, 0.32]	0.51
Ellipticity index of aorta	1.16 [1.11, 1.26]	1.21 [1.14, 1.29]	1.16 [1.11, 1.26]	0.62
Oversizing (diameter) (%)	20.0 [11.9, 28.1]	9.0 [5.13, 12.5]	20.5 [12.3, 28.3]	0.026
Oversizing (area) (%)	107.3 [67.9, 148.9]	58.1 [39.3, 74.1]	112.5 [68.7, 150.3]	0.035

^1^p-value according to Wilcoxon signed-rank test comparing patients with and without primary endoleaks

### Follow up

The median follow-up was 33 months [IQR 12 to 70]. Fifty (30.6%) patients had follow-up longer than five years. Survival follow-up was complete in 153 patients (96.8%) and clinical follow-up was fully updated in 133 patients (84.2%). Postoperative CTAs were available and of adequate quality among 144 patients (91.5%). 25 patients without updated clinical follow-up could not be contacted at the time of the study and had not attended a clinic visit six months prior. An average follow-up of 7 months [range 0–56 months] was available in these patients.

### Outcomes

**Fig. 3:**
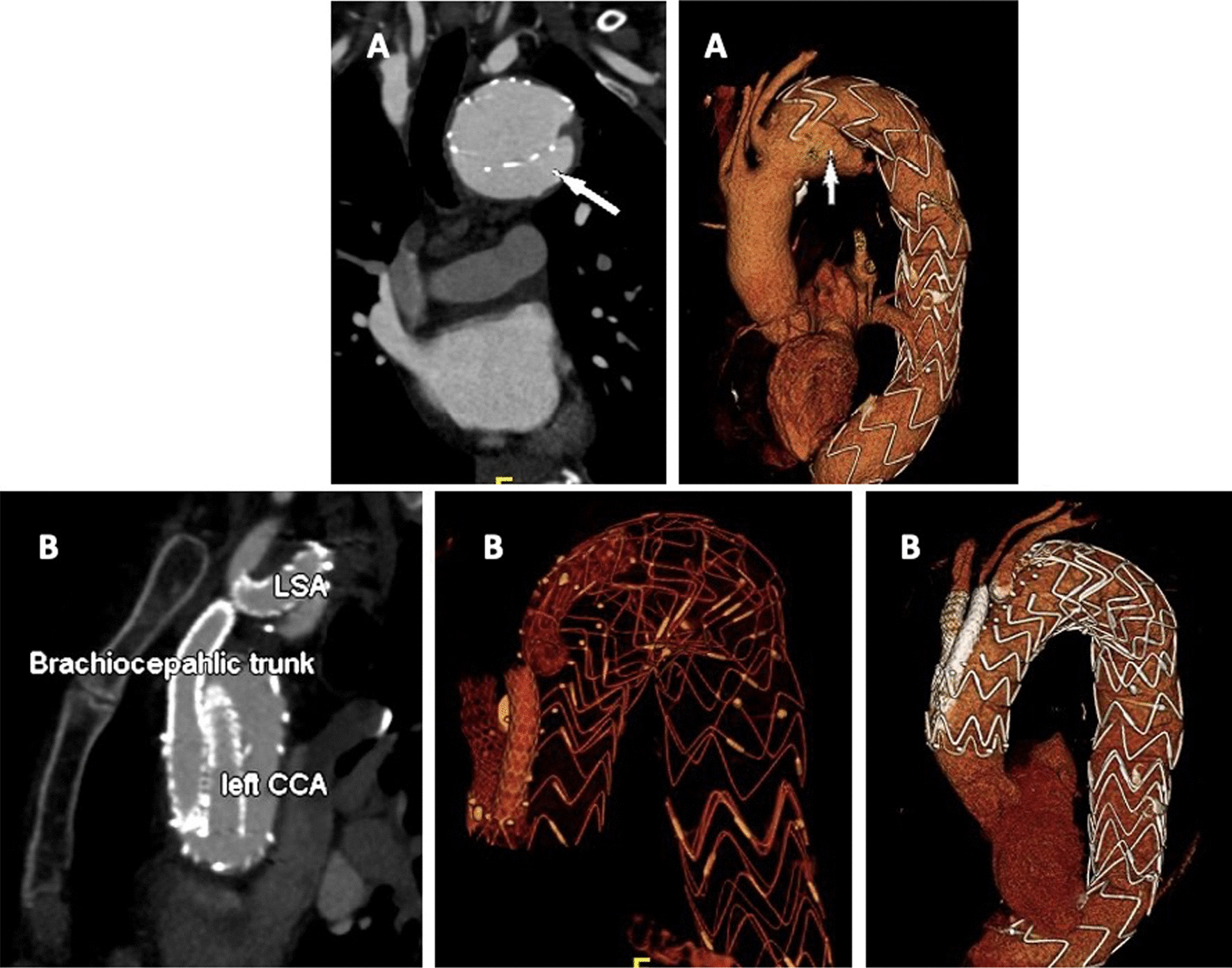
70 year-old female patient with type Ia endoleak 27 months after TEVAR (**A** arrow). The patient underwent total endovascular arch repair as proximal extention with a custom made triple branched stentgraft (retrograde branch for the LSA and antegrade branch for the left CCA and brachiocephalic trunk) as reintervention (**B**)

**Fig. 4:**
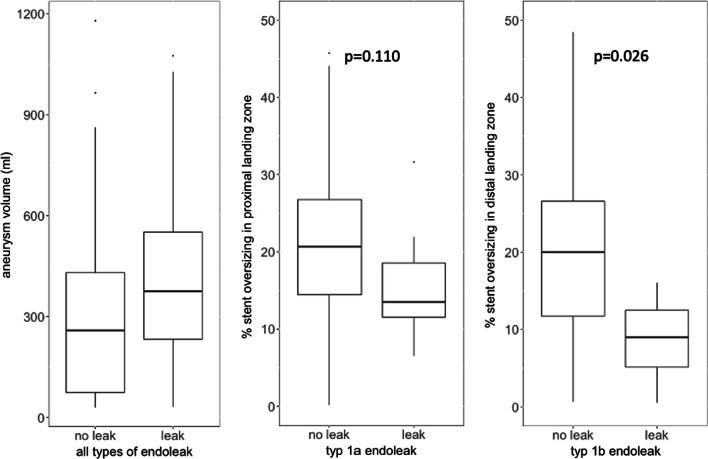
Aneurysm volume in patients with and without endoleak, proximal stent oversizing in patients with and without type Ia endoleak, and distal stent oversizing in patinets with and without type Ib endoleak

**Fig. 5:**
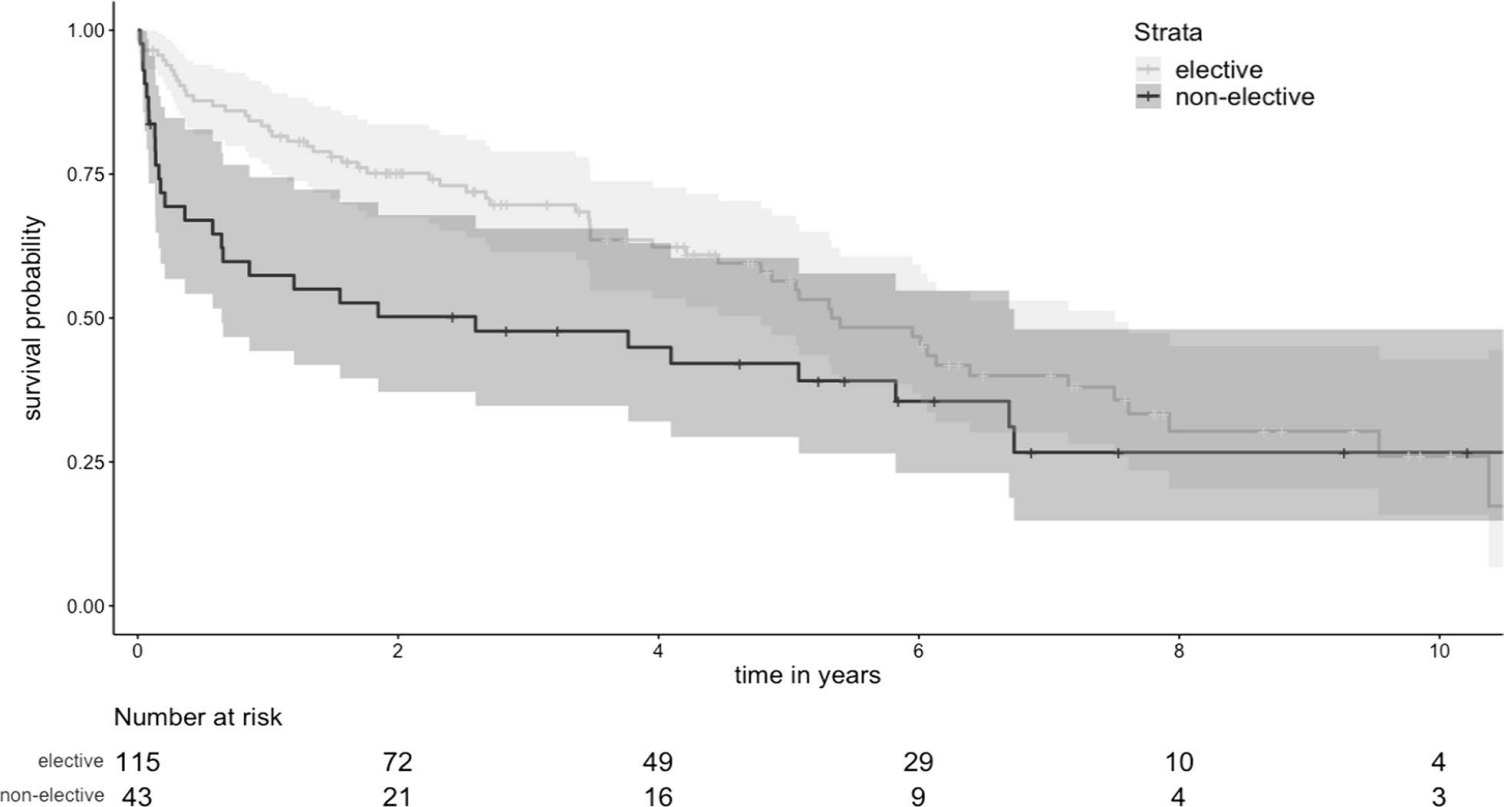
Kaplan–Meier estimate of survival in elective and non-elective cases

**Fig. 6:**
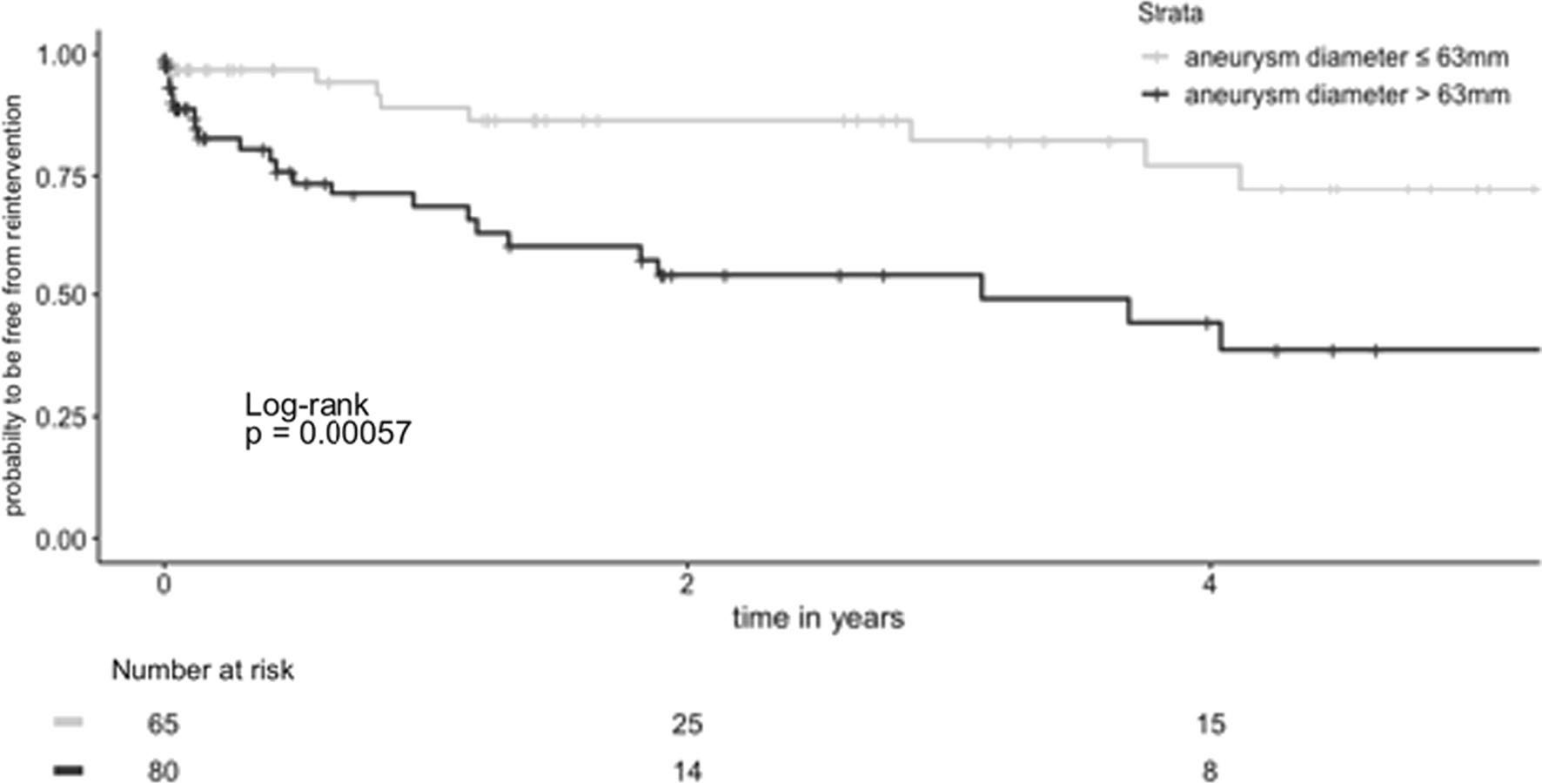
Probability to be free from aortic reintervention according to preoperative aneurysm diameter

Are presented in Tables 3 and 4 and illustrated in Figs. 2, 3, 4, 5 and 6.

**Table 3:** Outcomes

Patient outcomes (n = 158)	
30-day mortality	9 (5.6%)
Percentage of 30-day mortalities undergoing urgent TEVAR	5 (55.6%)
Respiratory failure	2 (1.3%)
Aneurysmal hemorrhage	1 (0.6%)
Cerebral hemorrhage	1 (0.6%)
Hemoptysis caused by aortobronchial fistula	1 (0.6%)
Unknown	4 (2.5%)
Postoperative stroke	4 (2.5%)
Spinal cord ischemia	0 (0)
Technical success*	133 (91.0%)
**Results from postoperative CTA (< 90 days, n = 144)**	
Type Ia endoleak	8 (5.6%)
Type Ib endoleak	4 (2.8%)
Type II endoleak	32 (22.2%)
From left subclavian artery	5 (3.5%)
From bronchial/intercostal arteries	27 (18.8%)
Type III endoleak	1 (0.7%)
**Overall Survival**	
One year	76.4% (70.0–83.3, SE 0.034%)
Five years	52.9% (45.0–62.2, SE 0.043%)
Ten years	26.8% (18.2–39.5, SE 0.053%)
Freedom of reintervention	
One year	80.0% (72.6–88.1, SE 0.039%)
Five years	52.8% (41.4–67.4, SE 0.065%)
Ten years	48.0% (35.3–65.3, SE 0.075%)

Data is reported as Kaplan–Meier estimate (95%CI) or n(%), *Technical success definded as the intraoperative survival of the patient, no intraoperative need for conversion to open surgical therapy and the absence of type I and type III endoleaks in the postoperative CTA

**Table 4:** Early and late reinterventions following TEVAR for DTA

Early reinterventions			
Reintervention (30 days)	n (%)	Indication	Time
Prox. Re-TEVAR w/o Debranching	2	Typ Ia endoleak	day 1 and 7
Prox. Re-TEVAR and LCCA-LSA	1	Polyaneurysmatic aorta	14 days
Distal. Re-TEVAR	2	Typ Ib endoleak	day 7 and 10
Amplatzer device in the LSA	2	Typ II endoleak	day 7 and 13
Balloon dilatation of stent	2	Incomplete apposition of stent	day 6 and 7
Implantation of a bare metal stent	1	Stent Kinking	8 days
Open aortic replacement of the ascending aorta	1	New dissection of the ascending aorta	2 days

Late reinterventions (longer than 30 days)	n (%)	Indication	Time
		Endoleak	
Prox. Re-TEVAR w/o Debranching	6	Typ Ia	at 2, 6, 14, 22, 30, 71 months
Distal Re-TEVAR	11	Typ Ib	at 3, 4, 5, 9, 10, 11, 15, 21, 23, 34, 48 months
Re-TEVAR	3	Typ III	at 14, 36, 47 months
Re-TEVAR	4	Aneruysm progression	at 2, 11, 47, 62 months
Re-TEVAR	1	New PAU	at 7 months
Open replacement	3	Infection	at 2, 6, 14 months

### 30-day outcomes

Survival at 30 days was 94.3% (95%CI 90.8–98.0, SE 0.018%), and known causes of death within the first 30 days are shown in Table 3. Four patients (2.5%) suffered postoperative stroke, two patients following TEVAR with total debranching procedure (n = 9) and two patients after TEVAR with LCCA-LSA (n = 61). There was no incident of spinal cord ischemia or phrenic nerve injury. Five patients (3.2%) had postoperative lower limb ischemia, of which three patients required surgical thrombectomy, one patient underwent iliac-femoral bypass and another patient symptomatically improved with anticoagulation and blood pressure management within a few days. Two (1.3%) patients developed an acute kidney injury. There were no postoperative myocardial infarctions.

### Survival

Survival probability was 76.4% (95%CI 70.0–83.3, SE 0.034%) at one year, 52.9% (95%CI 45.0–62.2, SE 0.043%) at five years and 26.8% (18.2–39.5, SE 0.053%) at 10 years.

In the first three years after TEVAR, survival among patients undergoing urgent or emergent TEVAR was lower than in elective cases (p = 0.002), but there was no significant difference beyond these first three years (p = 0.359) (Fig. 5). Though patients with acute presentation tended to have larger aneurysms than patients undergoing elective TEVAR (median aneurysm diameter 70.3 mm vs 63.4 mm, p = 0.032), the survival difference in the first three years was irrespective of aneurysm size (p = 0.075). Gender had no significant association with mortality (p = 0.395) or reintervention (p = 0.773).

On multivariable cox regression (*X*^2^ 31.43, p-value of the *X*^2^-test p < 0.001) using patient age, body mass index, left ventricular ejection fraction, New York Heart Association IV, estimated glomerular filtration rate (EGFR), Euroscore II, aortic diameter and thrombosed proportion of aneurysm, the following variables were significantly associated with mortality: A higher Euroscore II (HR 1.05, 95%CI 1.00–1.11, p = 0.046), aneurysm diameter (HR 1.02, 95%CI 1.00–1.04, p = 0.033), thrombosed proportion of aneurysm (HR 1.02, 95%CI 1.00–1.04, p = 0.032), and lower EGFR (HR 0.99, 95%CI 0.98–1.00, p = 0.048). Using maximally selected rank statistics to predict a cut-off value, an aneurysm diameter > 61 mm on the preoperative CTA was estimated to predict a higher probability of mortality during follow-up (HR 1.88, p = 0.021). Patients with an aneurysm diameter greater than 61 mm had a 1.88 increased risk of mortality compared to those with an aneurysm less than 61 mm in diameter). The estimated cut-off for EGFR was 63.3 ml/min and for 53.9% thrombosed proportion of aneurysm volume.

### Endoleaks

A CTA within the first three months was available in 144 (91.1%) patients. Rates of endoleaks are presented in Table 3. There were 8 (5.6%) type Ia endoleaks, 4 (2.8%) type Ib endoleaks and 32 (22.2%) type II endoleaks (Table 3). Type 1a and 1b endoleaks required reintervention while most of the type II endoleaks were self-resolved as detailed below and in Table 4. 20% of the patients without any sign of endoleak in their first postoperative CTAdeveloped new endoleaks after a median of 13.5 months [IQR 6 to 32] during follow-up. Late reinterventions included eleven patients (n = 6.9%) that required an intervention for type 1b endoleaks, partly resulting from aortic growth at distal landing zone (n = 5). Ten of the patients had Re-TEVAR as distal extension, and one patient received a bare metal stent. Patients with any type of endoleak during the first six months after TEVAR had significantly higher preoperative aneurysm length (Z = − 2.007, p = 0.045, r = 0.17) and aneurysm volume Z = 2.838, p = 0.025, r = 0.24) than those without endoleak. Patients with type Ib endoleak had significantly lower stent oversizing, both measured in diameter (Z = − 2.232, p = 0.026, r = 0.19) and area (Z = 2.115, p = 0.035, r = 0.18) than those without type Ib endoleak (Fig. 4). According to Youden's index, the diameter oversizing of 16.9% was the optimal cut off for the occurrence of an endoleak or not.

### Reintervention

In total, 24.8% (n = 39) patients received reintervention after a median of 7 months [IQR 0 to 22.5] following TEVAR, including five patients (12.8%) who needed more than one reintervention. There were 35 (76.9%) re-TEVAR and 4 (10.3%) open aortic replacements and 5 (12.8%) other reinterventions (implantations of bare metal stent, balloon dilatation of the stent, etc.). The indications for reintervention are detailed in Table 4. Patients with early type I endoleak were treated with Bare-metal stent (E-XL, Jotec) to achieve endoleak resolution, as illustrated in Fig. 3.

Patients with early endoleak showed a significantly higher rate of reintervention than patients without endoleak (p < 0.001). Overall Predictors for reintervention after TEVAR were larger aneurysm diameter (SHR 1.03, p = 0.002), and longer duration of procedure (SHR 1.01, p = 0.003), as an indicator for the complexity of the procedure. Using maximally selected rank statistics, an aneurysm diameter cut off of 62.7 mm on preoperative CTA was estimated. Patients with an aneurysm diameter greater than 63 mm were at 4.44 higher risk of reintervention compared to those with an aneurysm with a diameter below this threshold (p < 0.001).

Three patients (1.9%) suffered prosthesis infection at one month, eight months and 12 months post-operative, respectively. All three patients underwent open endograft removal and descending aortic replacement as well as appropriate antibiotic treatment, but all died in the ensuing weeks and months. Prosthesis infection was not associated with age (p = 0.678), BMI (p = 0.401), Euroscore (p = 0.199), duration of the procedure (p = 0.117) or vascular access type (p = 0.702).

### Proximal device landing zone and outcomes

Eighty patients required TEVAR with a LZ proximal to the left subclavian artery. The patients with LZ 0–2 had a significantly shorter LZ than those with LZ 3–4 (25.6 mm vs. 63.25 mm, p < 0.001). Patients with a very proximal LZ (0–1) were at 3.21 higher risk for long-term mortality (p = 0.003) and at 5.50 higher risk for later aortic reintervention (p = 0.009), compared to the rest of the cohort. LZ 2 was not associated with mortality (p = 0.686) or reintervention (p = 0.454), compared to the other patients in the cohort.

### Temporal analysis

There was no significant change over time in patient age (p = 0.882), aneurysm diameter (p = 0.936) and the proportion of patients that presented as acute cases (p = 0.257). However, the pre-operative Euroscore II (Kendall's tau-b correlation coefficient 0.131, p-value 0.026) (Additional file 1) and the proximal stent landing zones (Kendall's tau-b correlation coefficient − 0.195, p-value 0.001) (Additional file 2), significantly changed with time in that more patients with higher Euroscores and more proximal aneurysms underwent TEVAR more recently than prior. There was no significant difference in survival (p = 0.504), reintervention (p = 0.725) and endoleaks (p = 0.443) between patients in the first half of our study and the second half (before and after 2013).

## Discussion

After almost two decades of clinical application, TEVAR is a well-established therapy option for patients with descending thoracic aneurysms and recommended by current guidelines. [1] In our current study, with a moderately large cohort of 158 patients we analyzed the effects not only of patient characteristics such as aneurysm size and location, but also of the therapeutic approach such as oversizing, on midterm outcomes. Our cohort of patients treated from 2006 to 2019 in the era of modern thoracic stent-grafts systems, and improved pre- and intra-operative imaging, constitute the current real word application of TEVAR among DTA patients. 51% patients required proximal landing zones proximal to the left subclavian artery takeoff, and 25% underwent urgent or emergent TEVAR including 22.1% with aneurysm rupture. Compared to recent studies among DTA by Ranney et al. [11] and Ammar et al.[8], our cohort included patients with larger aneurysms (mean diameter 67 mm in our cohort vs 59 mm and 57 mm).

With a median follow-up of up 33 months, more than one third of the patients had a follow-up longer than five years. With a median patient age of 74 years, survival probability of 94.3%, at 30 days 76.4%, at one year and 52.9% at five years follow-up. This is comparable to prior studies evaluating outcomes of TEVAR for DTA. [11–15] Patients undergoing emergent or urgent TEVAR tended to have larger aneurysms than patients undergoing elective TEVAR (70.9 mm vs 65.9 mm, p = 0.032), supporting the notion of aortic surveillance and expectant intervention as recommended in the current practice guidelines. Irrespective of diameter requiring acute TEVAR was associated with lower survival probability in the first three post-operative years as demonstrated by Biancari et al. [12]. However, we additionally found that beyond these first few years, there was so significant difference between patients that were initially treated as emergent or elective case.

Overall, a proximal landing of the thoracic stent-graft in aortic zones 0–1 and larger aneurysm size were associated with both an increased risk for mortality and reintervention. Proximal landing in zones 0–1 was associated with 3 times increased risk for mortality and a 5.5 times increased risk for reintervention. Moreover, there were four additional patients that required aortic debranching for preparation of the proximal landing zone but then died before TEVAR, further demonstrating the increased risk associated with the requirement for very proximal landing zones. The correlation between large aneurysm size and proximal landing zones proximal to the LSA with higher risk following TEVAR for DTA was also demonstrated by Ammar et al*.* [7], Naazie et al.[16] and Chung et al*.* [17]. We additionally estimated cut offs of 61 mm and 63 mm aneurysm diameter correlated with a worse outcome regarding survival and the need of reintervention, respectively. Although large proximal aneurysms can be safely treated with TEVAR, there is still room for improvement. Of note, patients with larger aneurysms greater than 61 had a five-year survival of 50.6%, comparable to cohorts with smaller aneurysms that received open repair. [18]

In our series, 5.6% patients were noted to have primary type Ia endoleaks, and 2.8% primary type Ib endoleaks. In published series the rates of primary type I endoleaks are reported between 3.6% and 13.1%. [12, 14, 19, 20] In our study, there were 22% type II endoleaks with the majority originating from bronchial and intercostal arteries and self-resolved. To the contrary, most type II endoleaks from the subclavian artery required reintervention and did not self-resolve. The presence of an endoleak was associated with a higher risk of reintervention, but not with mortality. 20% of the patients without endoleak in their first postoperative CTA developed an endoleak during our follow-up, supporting the need for continuous aortic surveillance and subsequent interventions according to the guidelines.

We do not routinely use cerebrospinal fluid drainage catheters because of the risks associated with this intervention. In this cohort, no prophylactic drainage was applied in any of the patients. None of the patients suffered from spinal cord ischemia after the procedure.

Prosthetic infection is a feared complication after TEVAR and is difficult to manage. Jonker et al. [20] reported 2.3% of patients suffered from graft infections, occurring during the first months following the intervention. In most cases open surgery is necessary with poor outcomes. In our series prosthetic infections were affecting about 1.9% of the cohort. Due to excess mortality with and without surgery, primary prevention of prosthetic infection is of utmost importance.

## Limitations

The study is a single center retrospective study from the beginnings of the TEVAR therapy to modern times with more experience in this procedure. We also recognize that TEVAR continues to rapidly evolve, with additive improvements in clinical practice, perioperative care and devices during the past decade. An evaluation of the specific effect of these changes on outcomes was not possible. We focused on all-cause mortality instead of aorta-specific mortality as primary outcome because of the difficulty to arbitrate with 100% certainty. Further, follow-up CTA were not always available in the long-term as patients did not always return for the recommended follow-ups.

## Conclusion

This study reports real world outcomes following TEVAR for descending thoracic aneurysms. Although, the overall outcome of TEVAR for descending thoracic aneurysm is favorable, the large aneurysm size, acute presentation, proximal device landing zones 0–1, and early endoleaks are factors that all portend higher risks following TEVAR for thoracic aneurysms.

## Supplementary Information

**Additional file 1.** Analysis on the increase of the patients' Euroscore over the studys time span (using Kendall rank correlation coefficient).

**Additional file 2.** Analysis on the increase of more proximal landing zones over the studys time span (using Kendall rank correlation coefficient).

## Data Availability

The anonymized dataset used is available on reasonable request, within the auspices of the European general data protestation regulation 2020, for justifiable scientific purposes.
