# Supplementary figures and images for: Outcomes and survival following thoracic endovascular repair in patients with aortic aneurysms limited to the descending thoracic aorta
Source: J Cardiothorac Surg. 2023 Jun 20;18:194. doi: 10.1186/s13019-023-02285-3 (PMC10280975; doi:10.1186/s13019-023-02285-3)

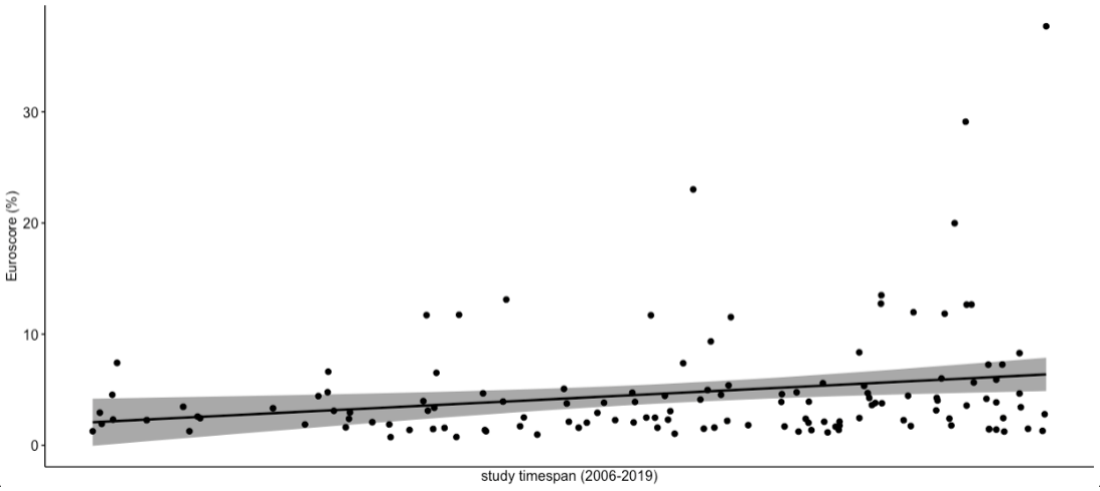

Supplement: Supplementary file 1 — Additional file 1. Analysis on the increase of the patients' Euroscore over the studys time span (using Kendall rank correlation coefficient). [file 13019_2023_2285_MOESM1_ESM.png]

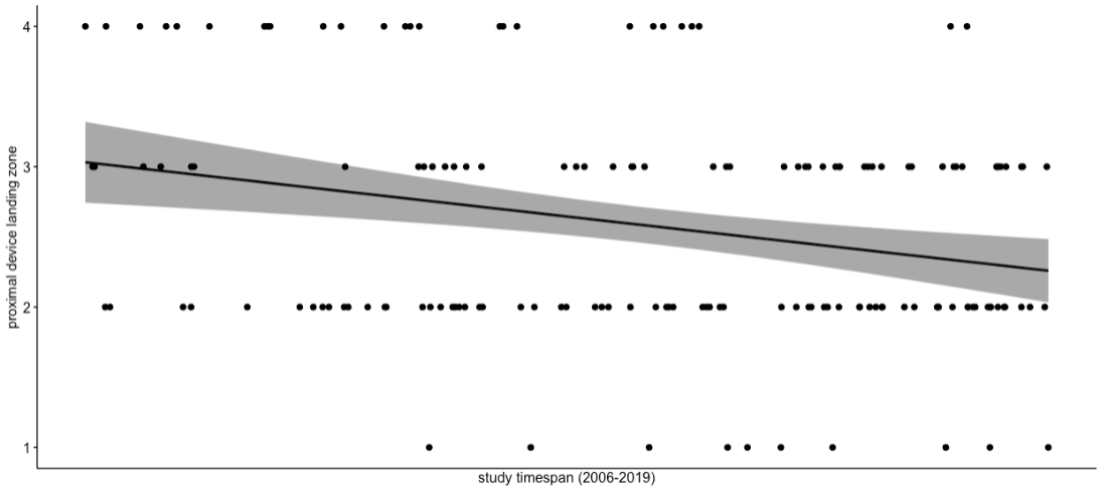

Supplement: Supplementary file 2 — Additional file 2. Analysis on the increase of more proximal landing zones over the studys time span (using Kendall rank correlation coefficient). [file 13019_2023_2285_MOESM2_ESM.png]
